# Sublethal exposure to *Microcystis aeruginosa* extracts during the yolk-sac larval stage reduces aerobic swimming speed in juvenile zebrafish

**DOI:** 10.1007/s10695-022-01151-8

**Published:** 2022-12-03

**Authors:** Athina Kekelou, Anastasia Dimitriadi, George Koumoundouros

**Affiliations:** Biology Department, University of Crete, Heraklion, Greece

**Keywords:** CyanoHABs, Fish, Yolk-sac larvae, Critical swimming speed

## Abstract

This study examined whether the aerobic swimming capacity of zebrafish juveniles is affected by the exposure of the yolk-sac larvae to sublethal concentration of *Microcystis aeruginosa* extract (200 mg dw L^−1^). Critical swimming speed significantly decreased in the pre-exposed fish (9.2 ± 1.0 vs 11.3 ± 1.4 TL s^−1^ in the control group). Exposure did not have any significant effects on the shape of the heart ventricle, rate of skeletal abnormalities, and growth or survival rates. Decreased swimming performance due to the early and short exposure to *M. aeruginosa* could have negative impacts on fish in the wild.

**Supplementary Information:**

The online version contains supplementary material available at 10.1007/s10695-022-01151-8.

## Introduction

Cyanobacterial harmful algal blooms (CyanoHABs) constitute a growing threat for ecosystems and public health (Paerl 2014). *Microcystis aeruginosa* is one of the predominant species in CyanoHABs that produces the endotoxin microcystins (MCs), together with a variety of other biologically active compounds (e.g., retinoids and oestrogenic compounds, Pipal et al. 2020; b-cyclocitral and b-Ionone, Li et al. 2021). *M. aeruginosa* (MA) extracts have high acute toxicity to the early life stages (ELS) of fish, mainly in the form of reduced survival and elevated malformation rates (Ghazali et al. 2009; Jonas et al. 2015; Saraf et al. 2018). At sublethal exposure levels, MA extracts may have less severe but persistent effects on developing fish. Sergi et al. (2022) demonstrated that exposure of zebrafish embryos (up to hatching, 48 h post-fertilization, hpf) to MA extracts resulted in decreased swimming performance, rounder heart ventricles, and elevated rates of vertebral abnormalities at later developmental stages.

Following our recent work on the prolonged effects of MA on zebrafish (Sergi et al. 2022), here, we hypothesized that exposure to sublethal levels of MA during the yolk-sac larval stage (from hatching to swimbladder inflation, 54–96 hpf) could also induce long-lasting effects in the swimming performance and anatomy (ventricular shape, skeleton abnormalities) of zebrafish juveniles.

## Materials and methods

### Zebrafish culture and exposure to *M. aeruginosa* extracts

Yolk-sac larvae were exposed by immersion to MA extract (200 mg biomass dw L^−1^), in glass beakers containing 150 larvae in 200 mL medium volume. Clean water was used for the control group. All trials were performed in two independent replicates. Following a 42-h exposure period, first-feeding larvae were reared up to the metamorphosis stage (22 dpf, ca 12–13 mm total length) in clean water, following the methodology of Sergi et al. (2022). In brief, larvae were reared into cubic net pens of 4.5 L volume each (0.1 mm mesh size), at 28.0 °C (± 0.5 °C), 86–95% oxygen saturation, 14/10 h light–dark photoperiod, 520–620 μS cm^−1^ conductivity, and 7.1–7.6 pH. To ensure common abiotic conditions for all experimental groups, pens were positioned into one common aquarium of 40 L volume, equipped with a biological filter. Larvae were fed five times daily with Artemia nauplii (Artemia AF, INVE, Determonde, Belgium) and commercial dry microdiets (Zebrafeed, Sparos Lda, Olhao, Portugal).

The selection of the tested extract concentration was based on the results of previous studies showing that 200 mg dw L^−1^ is the higher level that does not affect the survival rate of zebrafish embryos (Sergi et al. 2022). To test whether the selected level was not lethal for the early larval stage too, preliminary duplicated trials were performed by subjecting zebrafish yolk-sac larvae (54–96 hpf) to five extract concentrations (0, 50, 100, 200, 400 mg dw L^−1^). Fifty newly hatched yolk-sac larvae were used for each replicate and condition.

In all the trials, during the exposure period, oxygen saturation levels were controlled at normal levels by a gentle aeration of the medium, through pipette tips. The exposure medium was renewed twice daily. Before every medium renewal, dead embryos were removed, and oxygen levels, water temperature, and pH were measured. The effect of MA extract concentration on the measured abiotic conditions and fish survival rate was tested by means of the Kruskal–Wallis and Mann–Whitney *U* tests.

### Extract preparation

Cell-free crude MA extracts of lyophilized mass of *M. aeruginosa* (PCC 7806) culture were prepared as described in Sergi et al. (2022). Lyophilised MA mass was diluted in nanopure water and submitted to ultrasonic treatment on ice. The suspensions of broken cells were centrifuged, and the supernatants were stored at − 20 °C. Crude MA extracts contained 1.15 mg MCs L^−1^ at 200 mg biomass dw L^−1^ (Sergi et al. 2022).

### Swimming performance assay and heart morphology

Relative critical swimming speed (*RU*_crit_) was estimated by conducting incremental swimming tests, in a swimming apparatus composed of a swimming tunnel (70 cm length, 10 cm depth, 5 cm width) and two holding tanks (Koumoundouros et al. 2009). Different flow regimes were obtained using external magnetic pumps with adjustable valves. An electromagnetic flow meter (Valeport, Model 801) was used to calibrate water speed in the tunnel. After a 5-min acclimation period in static water, fish were exposed to an increasing water velocity (2 TL s^−1^ raise every 15 min), at 28 °C, until each individual was fatigued and unable to swim. After the swimming tests, fish were anesthetized (MS222), measured for TL, and fixed in buffered formalin. From each experimental group and replicate, 10–12 (22–24 in total) fish were tested for *RU*_crit_.

After swimming tests, formalin-fixed specimens (4–5 per experimental replicate, 9–10 per treatment) were stained with phosphomolybdic acid and examined for cardiac ventricle shape, by micro-CT imaging (SkyScan 1172, 2.0–2.5 μm pixel size, 50 kV voltage, 199 μA, 650 ms exposure time, 0.4° rotation step, 180° total rotation). Obtained projection images were then reconstructed to cross-sections and imported in the Amira v.5.2 software (Visage Imaging, Burlington USA). Ventricle morphometry was performed on the sagittal plane, as it was defined by the 1st vertebra, the ventral tip of cleithra, and the posterior of bulbus arteriosus. The ventricle length-to-depth ratio (VL/VD) was used for the measurement of ventricle roundness (Sergi et al. 2022).

The effect of MA exposure on the *RU*_crit_ and ventricle roundness was tested using the Mann–Whitney *U* test.

### Skeletal abnormalities and fish growth

Fish total length (TL) was measured at 22 dpf (days post-fertilization), on a random sample of 46–56 fish per replicate, individually anesthetized (2-phenoxyethanol, 0.2–0.3 mL L^−1^) and photographed. Fish samples were then stained for bone and cartilage (Walker and Kimmel 2007) and microscopically examined for the presence of skeletal abnormalities (e.g., deformations, missing elements, fusions). The effect of MA exposure on fish TL was tested using the Mann–Whitney *U* test. Differences in abnormality rates between the exposed and control groups were tested using *G*-test (Sokal and Rolhf 1981).

## Results and discussion

**Fig. 1:**
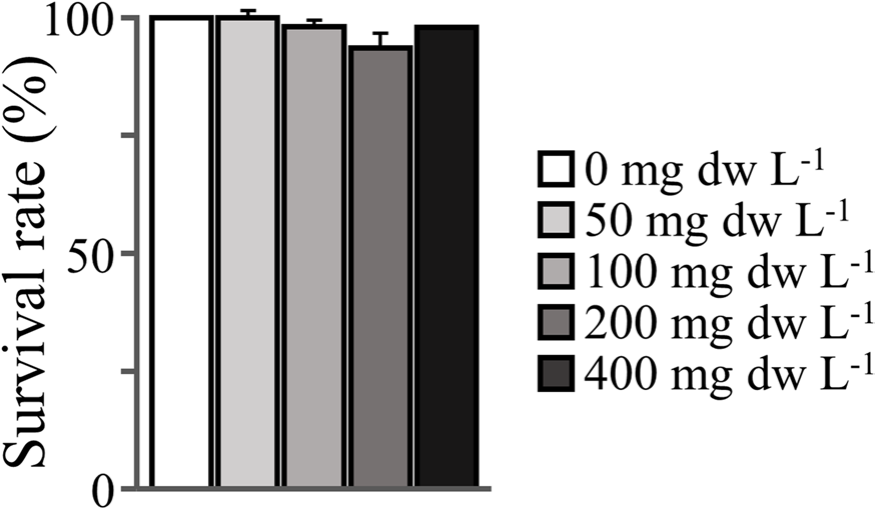
The effect of yolk-sac larval exposure to sublethal levels of *Microcystis aeruginosa* extracts on fish mean survival rate at the end of the exposure period (96 hpf). Error bars are equal to 1 SD. *n* = 2

Within the tested range, results indicated that MA extract concentration did not significantly affect fish survival (*p* > 0.05, Fig. 1), water oxygen concentration, and pH (Table S1).

**Fig. 2:**
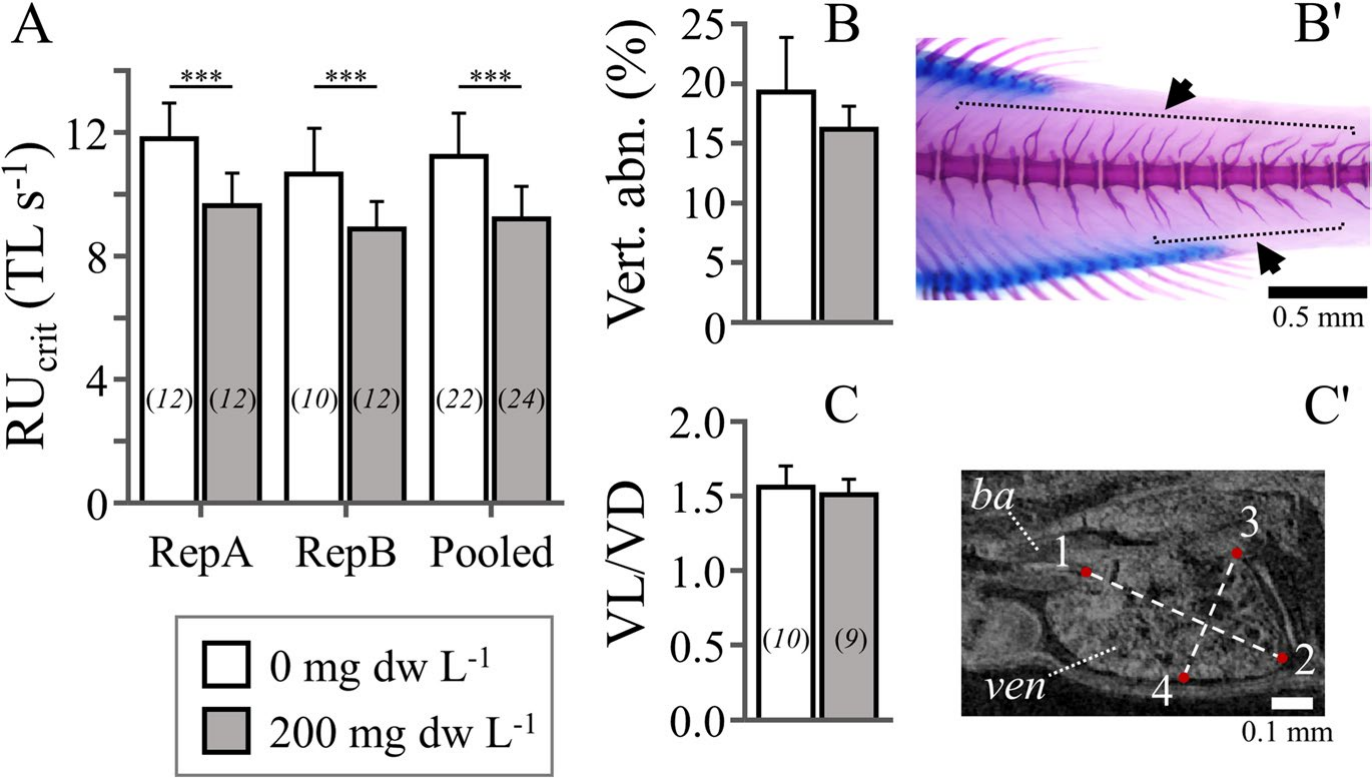
The effect of yolk-sac larval exposure to *M. aeruginosa* extract (200 mg dw L^*−*^.^1^) on the swimming performance and anatomy of zebrafish juveniles. **A** Critical swimming speed (*RU*_crit_), separately for each experimental replicate (RepA, RepB), or pooled. **B** Mean frequency of vertebral defects (arrows in **B'**). **C** Ventricle shape (VL/VD). **C'** Oblique slice showing the distance measurements taken (ven, ventricle; ba, bulbus arteriosus). One, ventriculo-bulbar valve. Two, apex. Three and 4 define the maximum ventricle depth (VD), perpendicularly to ventricle length (VL). Error bars = 1 SD. Numbers in parentheses (**A**, **C**) give the size of the samples. ***p < 0.001

Consistent with our initial hypothesis, pre-exposure to MA extract had a significant effect on fish swimming performance at late metamorphosis (*p* < 0.001). Compared with controls, MA-exposed fish presented a 17.9% decrease of the mean *RU*_crit_ (9.2 ± 1.0 in the pre-exposed vs 11.3 ± 1.4 TL s^−1^ in the control group, mean ± SD of the pooled data) (Fig. 2A). No significant differences existed in fish TL between the control and the exposed fish which were tested for *RU*_crit_ (Table S2). The observed decrease in *RU*_crit_ (present study) is similar to that reported by Sergi et al. (2022, 14.3%) to result from zebrafish exposure to MA during the embryonic stage.

**Fig. 3:**
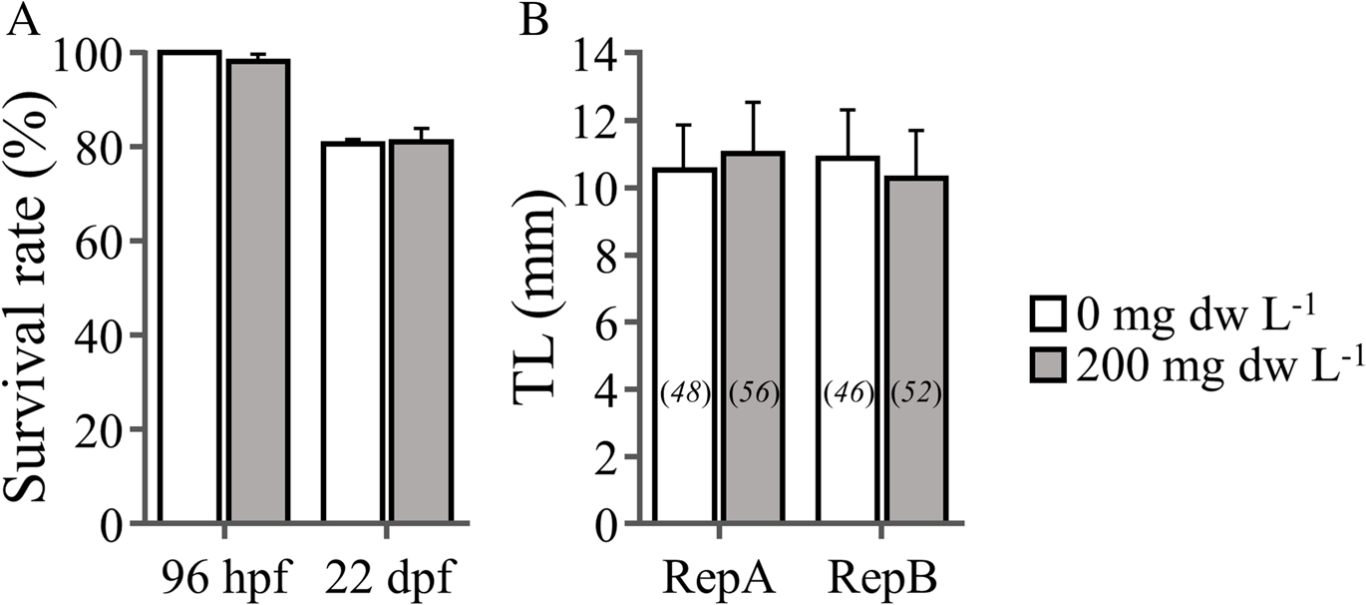
The effect of embryonic exposure to sublethal levels of *M. aeruginosa* extracts on fish survival and growth. **A** Survival rate at the end of the exposure period (96 hpf) and at the end of metamorphosis (22 dpf). **B** Total length at 22 dpf. Error bars are equal to 1 SD

Our initial hypothesis was rejected in the case of abnormalities frequency (Fig. 2B, B') and ventricle shape (Fig. 2C, C'), which presented no significant differences between the pre-exposed and control fish (*p* > 0.05). Detected abnormalities appeared mainly in the form of abnormal haemal and neural processes of the vertebral column (Fig. 2B'). Contrarily to our results, Sergi et al. (2022) showed that MA exposure during the embryonic stage resulted to an increased (by 11 times) abnormality rate and ventricle roundness (by 13.8%) (Table 1). Similarly to findings by Sergi et al. (2022), at the end of metamorphosis, no significant differences in survival rate and fish size were observed between the pre-exposed and the control groups (*p* > 0.05, Fig. 3A, B).

**Table 1:** Comparative responses (% change with respect to control) of zebrafish metamorphosing larvae to embryonic or yolk-sac larval exposure to *M. aeruginosa* crude extracts

	Embryonic exposure^1^	Yolk-sac larval exposure^2^
Exposure period (hpf, 28 °C)	1–48	54–96
Swimming performance	− 14.3%	− 17.9%
Ventricle roundness	+ 13.8%	ns
Vertebral abnormalities	+ 1071%	ns
Survival rate	ns	ns

^1^Sergi et al. (2022)

^2^Present study

To conclude, yolk-sac larval exposure to MA induced similar long-lasting effects on zebrafish swimming performance as in Sergi et al. (2022), but did not affect the ventricle shape and vertebral formation (Table 1). Despite the protective role of chorion, the sensitivity of zebrafish embryos to MA extracts (Table 1) might be linked to alterations of critical developmental processes, that take place before hatching and define fish development in the following stages. Somitogenesis and notochord differentiation in zebrafish take place before hatching (9–22 hpf, Kimmel et al. 1995), and, when defective, they result in the development of vertebral abnormalities during the following larval and juvenile period (Fleming et al. 2004; Lleras Forero et al. 2018). Similarly, long-lasting changes in ventricle shape (Sergi et al. 2022) might be linked with MA-induced changes of prior-to-hatching cardiac development events (e.g., chamber emergence, valvulogenesis, Glickman and Yelon 2002; Kalogirou et al. 2014). In the current study, *RU*_crit_ decrease in the MA pre-exposed fish was not linked to any cardiac-shape alterations or vertebral defects. Future studies could benefit from examining whether this decrease in *RU*_crit_ is linked to alterations of other features that are known to control aerobic swimming speed in fish (muscle physiology and/or functionality, mitochondria number, gill’s structure).

## Supplementary Information

Below is the link to the electronic supplementary material.

Supplementary file1 (DOCX 20 KB)

## Data Availability

The authors confirm that the data supporting the findings of this study are available within the article.

## References

[CR1] Fleming A, Keynes R, Tannahill D (2004). A central role for the notochord in vertebral patterning. Development.

[CR2] Ghazali IE, Saqrane S, Carvalho AP, Ouahid Y, Oudra B, Del Campo FF, Vasconcelos V (2009). Compensatory growth induced in zebrafish larvae after pre-exposure to a *Microcystis aeruginosa* natural bloom extract containing microcystins. Int J Mol Sci.

[CR3] Glickman NS, Yelon D (2002). Cardiac development in zebrafish: coordination of form and function. Semin Cell Dev Biol.

[CR4] Jonas A, Scholz S, Fetter E, Sychrova E, Novakova K, Ortmann J, Benisek M, Adamovsky O, Giesy JP, Hilscherova K (2015). Endocrine, teratogenic and neurotoxic effects of cyanobacteria detected by cellular *in vitro* and zebrafish embryos assays. Chemosphere.

[CR5] Kalogirou S, Malissovas N, Moro E, Argenton F, Stainier DYR, Beis D (2014). Intracardiac flow dynamics regulate atrioventricular valve morphogenesis. Cardiovasc Res.

[CR6] Kimmel CB, Ballard WW, Kimmel SR, Ullmann B, Schilling TF (1995). Stages of embryonic development of the zebrafish. Dev Dyn.

[CR7] Koumoundouros G, Ashton C, Xenikoudakis G, Giopanou I, Georgakopoulou E, Stickland N (2009). Ontogenetic differentiation of swimming performance in gilthead sea bream (*Sparus aurata*, Linnaeus 1758) during metamorphosis. J Exp Mar Biol Ecol.

[CR8] Li H, Gu X, Chen H, Mao Z, Zeng Q, Yang H, Kan K (2021). Comparative toxicological effects of planktonic *Microcystis* and benthic *Oscillatoria* on zebrafish embryonic development: implications for cyanobacteria risk assessment. Environ Pollut.

[CR9] Lleras Forero L, Narayanan R, Huitema LF, VanBergen M, Apschner A, Peterson-Maduro J, Logister I, Valentin G, Morelli LG, Oates AC, Schulte-Merker S (2018) Segmentation of the zebrafish axial skeleton relies on notochord sheath cells and not on the segmentation clock. eLife 7:e33843. 10.7554/eLife.33843.001 10.7554/eLife.33843 PMC5962341 29624170

[CR10] Paerl HW (2014) Mitigating harmful cyanobacterial blooms in a human- and climatically-impacted world. eLife 4:988–1012. 10.3390/elife4040988 10.3390/life4040988 PMC4284478 25517134

[CR11] Pipal M, Priebojova J, Koci T, Blahova L, Smutna M, Hilscherova K (2020). Field cyanobacterial blooms producing retinoid compounds cause teratogenicity in zebrafish embryos. Chemosphere.

[CR12] Saraf SR, Frenkel A, Harke MJ, Jankowiak JG, Gobler CJ, McElroy AE (2018). Effects of *Microcystis* on development of early life stage Japanese medaka (*Oryzias latipes*): comparative toxicity of natural blooms, cultured *Microcystis* and microcystin-LR. Aquat Toxicol.

[CR13] Sergi E, Orfanakis M, Dimitriadi A, Christou M, Zachopoulou A, Kourkouta Ch, Printzi A, Zervou S-K, Makridis P, Hiskia A, Koumoundouros G (2022). Sublethal exposure to *Microcystis aeruginosa* extracts during embryonic development reduces aerobic swimming capacity in juvenile zebrafish. Aquat Toxicol.

[CR14] Sokal R, Rohlf F (1981) Biometry. 2nd ed. WH Freeman, San Francisco, California, USA.

[CR15] Walker MB, Kimmel CB (2007). A two-color acid-free cartilage and bone stain for zebrafish larvae. Biotech Histochem.

