# Supplementary material for: Sublethal exposure to Microcystis aeruginosa extracts during the yolk-sac larval stage reduces aerobic swimming speed in juvenile zebrafish
Source: Fish Physiol Biochem. 2022 Dec 3;48(6):1443–7. doi: 10.1007/s10695-022-01151-8 (PMC9763139; doi:10.1007/s10695-022-01151-8)
Supplement: Supplementary file 1 — Supplementary file1 (DOCX 20 KB) [file 10695_2022_1151_MOESM1_ESM.docx]

Sublethal exposure to *Microcystis aeruginosa* extracts during the yolk-sac larval stage reduces aerobic swimming speed in juvenile zebrafish

Athina Kekelou, Anastasia Dimitriadi, George Koumoundouros^*^

Biology Department, University of Crete, Heraklion, Greece

* author for correspondence, [gkoumound@uoc.gr](mailto:gkoumound@uoc.gr) (ORCID: 0000-0002-9738-0403)

**Table S1**. Abiotic conditions during the exposure period (54-96 hpf) of the preliminary (sublethal levels estimation) and the main exposure trials. In each replicate, extract concentration had no significant effect on the abiotic conditions (p>0.05, Kruskal-Wallis or Mann-Whitney U test).

| Trial | Replicate | Extract concentration (mg dw L^-1^) | Ο_2_ (mg L^-1^) | pH | Temperature (^ο^C) |
| --- | --- | --- | --- | --- | --- |
| Sublethal levels estimation | Α | 0 | 7.5±0.2 | 7.8±0.2 | 27.7±0.4 |
|  |  | 50 | 7.4±0.3 | 7.7±0.2 | 27.7±0.4 |
|  |  | 100 | 7.3±0.4 | 7.7±0.2 | 27.7±0.4 |
|  |  | 200 | 7.1±0.5 | 7.7±0.2 | 27.7±0.4 |
|  |  | 400 | 7.1±0.6 | 7.7±0.1 | 27.7±0.4 |
|  | Β | 0 | 7.6±0.2 | 8.0±0.2 | 28.0±0.2 |
|  |  | 50 | 7.5±0.3 | 7.9±0.2 | 28.0±0.2 |
|  |  | 100 | 7.5±0.2 | 7.9±0.1 | 28.0±0.2 |
|  |  | 200 | 7.4±0.3 | 7.9±0.2 | 28.0±0.2 |
|  |  | 400 | 7.3±0.4 | 8.0±0.3 | 28.0±0.2 |
| Main | Α | 0 | 7.3±0.3 | 7.5±0.3 | 28.0±0.2 |
|  |  | 200 | 7.1±0.3 | 7.5±0.2 | 28.0±0.2 |
|  | Β | 0 | 7.5±0.2 | 7.3±0.1 | 28.0±0.2 |
|  |  | 200 | 7.2±2.5 | 7.3±0.3 | 28.0±0.2 |

**Table S2**. Mean total length (TL) of zebrafish studied for critical swimming speed. In each replicate, fish TL did not differ significantly between the different treatments (p>0.05, Mann-Whitney U test).

| Replicate | Extract concentration (mg dw L^-1^) | TL  (±SD, mm) | n |
| --- | --- | --- | --- |
| Α | 0 | 12.6±1.2 | 12 |
|  | 200 | 12.7±0.7 | 12 |
| Β | 0 | 12.1±0.8 | 10 |
|  | 200 | 12.6±1.1 | 12 |
| Pooled | 0 | 12.4±1.0 | 22 |
|  | 200 | 12.7±0.9 | 24 |
